# Etiology of tricuspid regurgitation and mortality: a multicenter cohort study

**DOI:** 10.1007/s00392-025-02662-z

**Published:** 2025-05-08

**Authors:** Jorge Rodríguez-Capitán, Paloma Márquez-Camas, Jesús Carmona-Carmona, Diego Félix Arroyo Moñino, Marinela Chaparro-Muñoz, Matías Soler-González, Manuel García del Río, Teodora Egido de la Iglesia, Jorge Segovia-Reyes, Mora Murri, José Raúl López Salguero, David Couto-Mallón, Miguel Romero-Cuevas, Francisco Javier Pavón-Morón, Mario Gutiérrez-Bedmar, Manuel Jiménez-Navarro

**Affiliations:** 1 Instituto de Investigación Biomédica de Málaga y Plataforma en Nanomedicina (IBIMA Plataforma BIONAND), Málaga, Spain; 2 Área del Corazón, Hospital Universitario Virgen de la Victoria, Málaga, Spain; 3 Centro de Investigación Biomédica en Red de Enfermedades Cardiovasculares (CIBERCV), Instituto de Salud Carlos III, Madrid, Spain; 4 Servicio de Cardiología, Hospital Universitario Virgen Macarena, Seville, Spain; 5 Área Quirúrgica, Hospital De Antequera, Antequera, Spain; 6 Servicio de Cardiología, Hospital Punta de Europa, Algeciras, Spain; 7 IES Federico García Bernalt, Salamanca, Spain; 8 Endocrinolgy and Nutrition UCG, Hospital Universitario Virgen de La Victoria, Málaga, Spain; 9 Centro de Investigación Biomédica en Red en Fisiopatología de Obesidad y Nutrición (CIBERObn), Instituto de Salud Carlos III, Madrid, Spain; 10 Servicio de Cardiología, Hospital de Antequera, Antequera, Spain; 11 Servicio de Cardiología, Complexo Hospitalario Universitario de A Coruña, A Coruña, Spain; 12 Instituto de Investigación Biomédica de A Coruña (INIBIC), A Coruña, Spain; 13 Departmento de Medicina Preventiva y Salud Pública, Facultad de Medicina, Universidad de Málaga, Málaga, Spain; 14 Departamento de Medicina y Dermatología, Facultad de Medicina, Universidad de Málaga, Málaga, Spain

**Keywords:** Tricuspid regurgitation, Mortality, Etiology, Prognosis

## Abstract

**Background:**

Significant tricuspid regurgitation (TR) encompasses a wide range of etiologies, complicating a comprehensive understanding of disease progression and prognostic factors. This study aimed to assess mortality associated with significant TR, focusing on the role of valvular disease etiology and other predictive factors.

**Methods:**

This is a retrospective, multicenter, cohort observational study, including all consecutive patients with moderate-to-severe or greater TR. The patients were classified into five etiological groups: organic TR, TR secondary to left valvulopathy, TR secondary to left or right ventricular dysfunction, TR secondary to pulmonary hypertension, and atrial TR. The long-term mortality was assessed (median follow-up: 39.8 months).

**Results:**

757 patients were included. The overall mortality incidence rate was 162.5 deaths per 1000 patient-years. Compared to atrial TR, all other etiologies presented a higher mortality risk: organic TR adjusted hazard ratio (aHR) = 2.344 (95% confidence interval [CI]: 1.138–4.829), left valvulopathy-related TR aHR = 1.901 (95% CI: 1.011–3.574), ventricular dysfunction-related TR aHR = 3.683 (95% CI: 1.627–8.338), and pulmonary hypertension-related TR aHR = 2.446 (95% CI: 1.215–4.927). In addition to known factors, male sex was associated with a higher mortality risk (aHR = 1.608, 1.175–2.201), while beta-blocker use was linked to a lower risk (aHR = 0.674, 0.502–0.904).

**Conclusions:**

In a large cohort of patients with significant TR, and after adjusting for clinical and echocardiographic variables, all etiological groups exhibited a higher mortality risk compared to atrial TR. Additionally, male patients with TR had a higher mortality risk, while beta-blocker therapy emerged as a protective factor.

## Introduction

Significant tricuspid regurgitation (TR) is a condition of recognized importance due to its high prevalence and its association with elevated morbidity and mortality [1, 2]. One of the major challenges in understanding TR is its etiological heterogeneity. Various research groups have delved into the pathophysiology of different types of TR, describing their distinct causes and mechanisms, even proposing new subgroups such as atrial [3]. Additionally, when each etiological group is analyzed individually, it has been consistently linked to poor prognosis [2, 4].

However, these findings have not significantly impacted the morbidity and mortality outcomes of patients, as the current evidence demonstrating the prognostic benefit of therapeutic tools for TR (medical treatment, surgery, and percutaneous intervention) is minimal [5, 6]. These knowledge gaps highlight the need for a deeper understanding of the specific natural history of different etiological types of TR to better comprehend their clinical evolution and prognostic factors.

Therefore, the objective of this study was to evaluate the clinical prognosis, in terms of mortality, of a large cohort of patients with significant TR, comparing different etiological types of TR to assess the specific prognostic significance of each. Additionally, we aimed to identify other independent factors related to the mortality of these patients.

## Methods

### Study population and design

This was an observational, retrospective cohort study conducted at three centers in Spain, including two tertiary hospitals and one secondary hospital. The inclusion criteria were as follows: (a) moderate-severe or greater TR on a native valve documented in at least one echocardiogram performed between January 1, 2002, and December 31, 2017 (inclusion period), in both inpatients and outpatients, and (b) age ≥ 18 years. The exclusion criteria were: (a) prior tricuspid valve surgery or percutaneous intervention before study inclusion, (b) moderate-severe or greater TR documented in the medical record before the inclusion period, and (c) congenital TR.

The patients were consecutively included after a manual review of all echocardiogram reports issued during the inclusion period. Due to missing data, one center included patients from January 2015 to December 2017, and other center from March 2005 to December 2017, as they only had access to all consecutive reports from echocardiographic studies during these intervals. The study was initiated after approval by the Provincial Ethics Committee (project code 1413-N-18, approval date 06/10/2018).

### Baseline echocardiographic and clinical variables

The echocardiographic studies were performed by experienced cardiologists, following the recommendations for the echocardiographic evaluation of valvular heart disease applicable during the different periods of the study. The severity of TR was determined based on a comprehensive assessment using qualitative and semi-quantitative parameters of the regurgitant jet, tricuspid valve morphology, and the dimensions of the right atrium and right ventricle [7–9]. TR severity was classified based on the report recorded in the medical history into three categories: moderate-severe (if parameters fell between moderate and severe), severe, and more-than- severe (if a free regurgitant jet without valvular opposition was observed).

The patients were classified according to the type of TR based on their clinical context, as derived from the medical records. For this, we used the stepwise classification proposed by Topilsky et al. [1], in which TR is defined according to the first step that meets the established criteria, regardless of whether the criteria for subsequent categories were also met. In the first step, congenital TR was defined as TR caused by any congenital heart disease, whether corrected or not. In the second step, organic TR was defined as non-congenital TR associated with structural disease of the tricuspid valve (including confirmed or possible interference by a pacemaker lead). In the third step, functional TR associated with left-sided valve disease was defined as non-congenital, non-organic TR occurring in patients with left-sided valve prostheses, previous surgical or percutaneous left-sided valve repair, any degree of mitral stenosis, or any other moderate or greater left-sided valve disease. In the fourth step, functional TR associated with left ventricular (LV) systolic dysfunction or primary right ventricular (RV) disease was defined as non-congenital, non-organic, non-left-sided valve disease observed in patients with LV systolic dysfunction (ejection fraction [EF] < 50%) or intrinsic RV structural pathology. In the fifth step, functional TR associated with pulmonary hypertension was defined as TR not meeting the criteria for the previous four steps but with a systolic pulmonary artery pressure (SPAP) ≥ 50 mmHg. In the sixth step, atrial TR was defined as TR not meeting any previous step criteria and presenting with any degree of right atrial (RA) dilation and SPAP < 50 mmHg. Finally, TR of unknown origin was defined as TR that did not meet the criteria for any of the previous categories. For this study, six comparison groups were defined according to TR etiology, as the first described group (congenital etiology) was excluded.

Regarding other echocardiographic variables, only those recorded in the medical records of a high percentage of patients were included, following recommendations in clinical guidelines during the echocardiographic studies [7–10]: tricuspid stenosis, SPAP, LV, RV, and RA dilation, presence and degree of LV hypertrophy, LV systolic function, and presence of RV systolic dysfunction. SPAP was estimated based on the maximum velocity of TR flow in systole, according to the Bernoulli equation, adding the estimated pressure for the RA. LV systolic function was classified as preserved (EF ≥ 50%), mild dysfunction (EF 40–49%), moderate dysfunction (EF 30–39%), or severe dysfunction (EF < 30%). LV dilation and the degree of LV hypertrophy (absent, mild, moderate, or severe) were defined according to clinical guidelines at the time. RV dilation and/or dysfunction were assessed qualitatively. RV dilation was considered present if, in the standard apical 4-chamber view, the RV appeared larger than 2/3 of the size of the LV or if the RV occupied the apex, displacing the LV. RV systolic dysfunction was defined by visual estimation after evaluation in multiple views. RA dilation was also defined qualitatively by comparing the size of the RA to other cardiac chambers in standardized views. The baseline clinical variables were also collected from medical records.

### Follow-up

For each patient, the date of the first echocardiogram showing moderate-severe or greater TR within the inclusion period was considered the start of follow-up. Follow-up continued until death or August 31, 2022. The primary study outcome was all-cause mortality, recorded from the patients’ medical records and the National Death Index. Data on tricuspid valve surgery during follow-up and perioperative mortality were also obtained from medical records.

### Statistical analysis

Baseline clinical and echocardiographic characteristics were summarized using the mean and 95% confidence interval (95% CI) for continuous variables and compared across etiological groups using ANOVA. The categorical variables were expressed as counts and percentages and compared by etiological group using the Chi-square test. The follow-up time was measured in months and reported as the median and interquartile range. The percentage of patients undergoing cardiac surgery in each group was compared using the Chi-square test.

The incidence rate per 1000 patient-years, along with the 95% CI, was calculated for each etiological group, and the rate ratio comparing each group to the rest was also determined. These analyses were repeated separately by sex. Kaplan–Meier survival curves for the different etiological groups were compared using the log-rank test.

The association between the studied variables and mortality was analyzed using adjusted Hazard Ratios (aHR) and their 95% CI, estimated through a Cox regression model, with mortality as the dependent variable. Variables recognized as predictors in the literature, along with those associated with mortality in univariate analysis with a *p*-value < 0.25, were included as independent variables. The final model included the following variables: etiological group (reference: atrial TR), age (years), sex (reference: male), TR grade (reference: moderate-severe TR), SPAP (continuous), LV hypertrophy (reference: absent), NYHA functional class (reference: class I), and the following dichotomous variables (present/absent, with absent as the reference category): previous valve surgery or intervention, RV dilation, RV dysfunction, RA dilation, diabetes, chronic kidney disease, liver disease, lung disease, ischemic heart disease, prior heart failure (HF) hospitalization, hemoglobin < 11 g/dl, right-sided HF, diuretic treatment and beta-blocker treatment.

## Results

### Baseline characteristics

A total of 757 patients with moderate-to-severe or greater TR were included. The most frequent group was TR associated with left-sided valvular disease, representing 57.1% of the cohort, followed by functional TR associated with PH (18.4%), organic TR (11.0%), atrial TR (8.5%), and functional TR associated with LV systolic dysfunction or primary RV disease (5.2%). No cases were classified as TR of unknown origin. Table 1 provides a detailed breakdown of the etiology within each of the four main TR groups. Among the 39 patients in the group with TR associated with LV systolic dysfunction or primary RV disease, only three presented primary RV involvement (without LV dysfunction). Table 2 shows the baseline characteristics across the five TR types at diagnosis. Notably, 71.2% of the overall sample were women. This female predominance was observed in four of the five groups, with the exception of the functional TR associated with LV or RV dysfunction group, where only 43.6% were women.

**Table 1:** Description of the predominant etiology of TR in each of the groups

*Organic TR (n = 83)*	
Degenerative	6 (7.2%)
Endocarditis	6 (7.2%)
Carcinoid tumor	2 (2.4%)
Cardiac pacing device-related	38 (45.8%)
Rheumatic tricuspid involvement	31 (37.3%)
*Functional TR associated with left-sided valve disease (n = 432)*	
*According to the type of left-sided valve disease*	
Isolated mitral valvulopathy	242 (56.0%)
Isolated aortic valvulopathy	61 (14.1%)
Mitral and aortic valvulopathy	129 (29.9%)
*According to the status of the left-sided valve disease*	
Previously corrected left-sided valvulopathy	281 (65.1%)
Uncorrected left-sided valvulopathy	151 (35.0%)
*Functional TR associated with LV systolic dysfunction or primary RV disease (n = 39)*	
LV systolic dysfunction	36 (92.3%)
Primary RV disease	3 (7.7%)
*Functional TR associated with pulmonary hypertension (n = 139)*	
PH Group 1	5 (3.6%)
PH secondary to heart failure with preserved ejection fraction	15 (10.8%)
PH Group 3 and/or Group 4	99 (71.2%)
PH of unknown etiology	20 (14.4%)

The data represent the total number of patients for each specific etiology and the percentage relative to the group to which they belong

*LV* left ventricular, *PH* pulmonary hypertension, *RV* right ventricular, *TR* tricuspid regurgitation

**Table 2:** Baseline characteristics of patients, comparing by TR type

	Organic TR (*n* = 83, 11.0%)	Functional TR associated with left-sided valve disease (*n* = 432, 57.1%)	Functional TR associated with LV systolic dysfunction or primary RV disease (*n* = 39, 5.2%)	Functional TR associated with pulmonary hypertension (*n* = 139, 18.4%)	Atrial TR (*n* = 64, 8.5%)	*p*
Sex (female)	69.9%	72.0%	43.6%	74.1%	78.1%	0.002
Age (years), Mean (95%CI)	70.8 (68.3–73.3)	73.2 (72.1–74.3)	68.6 (65.0–72.3)	73.4 (71.5–75.3)	76.6 (73.8–79.4)	0.005
*TR grade*						0.137
Moderate-severe TR	33.7%	47.0%	53.9%	47.5%	54.7%	
Severe TR	60.2%	50.7%	43.6%	47.5%	40.6%	
More than severe TR	6.0%	2.3%	2.6%	5.0%	4.7%	
Tricuspid Stenosis	26.5%	0	0	0	0	< 0.001
SPAP (mmHg), Mean (95%CI)	46.0 (42.8–49.3)	55.1 (53.7–56.4)	51.4 (46.8–56.1)	69.7 (67.3–72.0)	42.4 (38.9–45.9)	< 0.001
Previous cardiac surgery or valve intervention	24.1%	35.0%	0	0	0	< 0.001
LV dilation	10.8%	20.2%	41.0%	0.7%	0	< 0.001
*LV systolic function*						< 0.001
Preserved	83.1%	78.2%	7.7%	100%	100%	
Mild dysfunction	7.2%	4.9%	23.1%	0	0	
Moderate dysfunction	4.8%	7.9%	28.2%	0	0	
Severe dysfunction	4.8%	9.0%	41.0%	0	0	
*LV hypertrophy*						0.34
Absent	80.5%	66.0%	69.2%	69.3%	73.4%	
Mild	13.4%	27.6%	23.1%	25.6%	18.8%	
Moderate	4.9%	4.9%	5.1%	5.1%	7.8%	
Severe	1.2%	1.4%	2.6%	0	0	
RV dilation	73.8%	66.8%	81.1%	78.5%	61.7%	0.023
RV systolic dysfunction	13.7%	21.4%	55.6%	25.0%	11.1%	< 0.001
Right atrial dilation	87.8%	83.5%	88.9%	85.4%	100%	0.010
Atrial fibrillation	86.8%	87.6%	78.4%	71.1%	89.1%	< 0.001
Hypertension	67.1%	67.1%	68.4%	71.6%	75.0%	0.691
Diabetes	35.8%	34.3%	32.4%	36.8%	20%	0.208
Dyslipidemia	22.2%	28.2%	29.7%	25.8%	28.3%	0.823
Smoking	11.4%	12.7%	21.6%	18.9%	13.6%	0.247
Chronic kidney disease	30.5%	35.9%	43.2%	40.2%	41.0%	0.511
Liver disease	9.9%	10.2%	13.5%	9.2%	5.0%	0.685
OSAS	0	6.2%	10.8%	22.7%	10.0%	< 0.001
Lung disease	9.9%	19.6%	21.1%	32.3%	16.7%	0.001
Ischemic heart disease	15.9%	16.5%	40.5%	9.9%	16.4%	0.001
Previous hospitalization for heart failure	70.4%	65.2%	74.4%	63.0%	36.7%	< 0.001
Hemoglobin < 11 g/dL	32.8%	29.7%	30.0%	38.2%	30.6%	0.589
Platelets < 90.000 per mcL	4.0%	3.6%	7.1%	5.4%	0	0.579
*NYHA functional class*						< 0.001
NYHA I	6.7%	7.5%	3.0%	7.3%	17.7%	
NYHA II	48.3%	41.3%	24.2%	29.4%	47.1%	
NYHA III	41.7%	43.4%	42.4%	45.9%	29.4%	
NYHA IV	3.3%	7.8%	30.3%	17.4%	5.9%	
Right heart failure	63.3%	65.8%	81.6%	72.9%	68.3%	0.169
Diuretics	82.6%	79.7%	73.5%	75.6%	76.9%	0.698
Beta-blockers	46.3%	41.2%	61.8%	31.1%	40.4%	0.020
ACEi/ARBs	53.7%	51.5%	55.9%	47.9%	53.9%	0.890
Aldosterone antagonists	23.5%	23.3%	41.2%	19.3%	9.6%	0.013

*ACEi* Angiotensin-Converting Enzyme inhibitors, *ARBs* Angiotensin II Receptor Blockers, *CI* confidence interval, *LV* left ventricular, *NYHA* New York Heart Association, *OSAS* Obstructive Sleep Apnea Syndrome, *RV* right ventricular, *SPAP* systolic pulmonary artery pressure, *TR* tricuspid regurgitation

### Follow-up of the total sample and subgroups

The median clinical follow-up duration for total mortality evaluation was 39.8 months (interquartile range [IQR]: 11.0–80.2 months). 3.2% of participants were censored due to incomplete follow-up. Only 11.7% of patients who were not lost to follow-up underwent tricuspid valve surgery, with a median time to surgery of 18.8 months (IQR: 3.3–57.3 months). Table 3 reports the percentage of patients who underwent surgery across the five groups, with significant differences observed (*p* < 0.001). Perioperative mortality was 16.3%.

**Table 3:** Percentage of patients undergoing tricuspid surgery in each group

Group	Percentage (%)	*p* < 0.001
Organic	22.2	
Functional associated with left-sided valve disease	15.1	
Functional associated with LV systolic dysfunction or primary RV disease	5.6	
Functional associated with pulmonary hypertension	1.5	
Atrial	1.6	

*LV* left ventricular, *RV* right ventricular

**Fig. 1:**
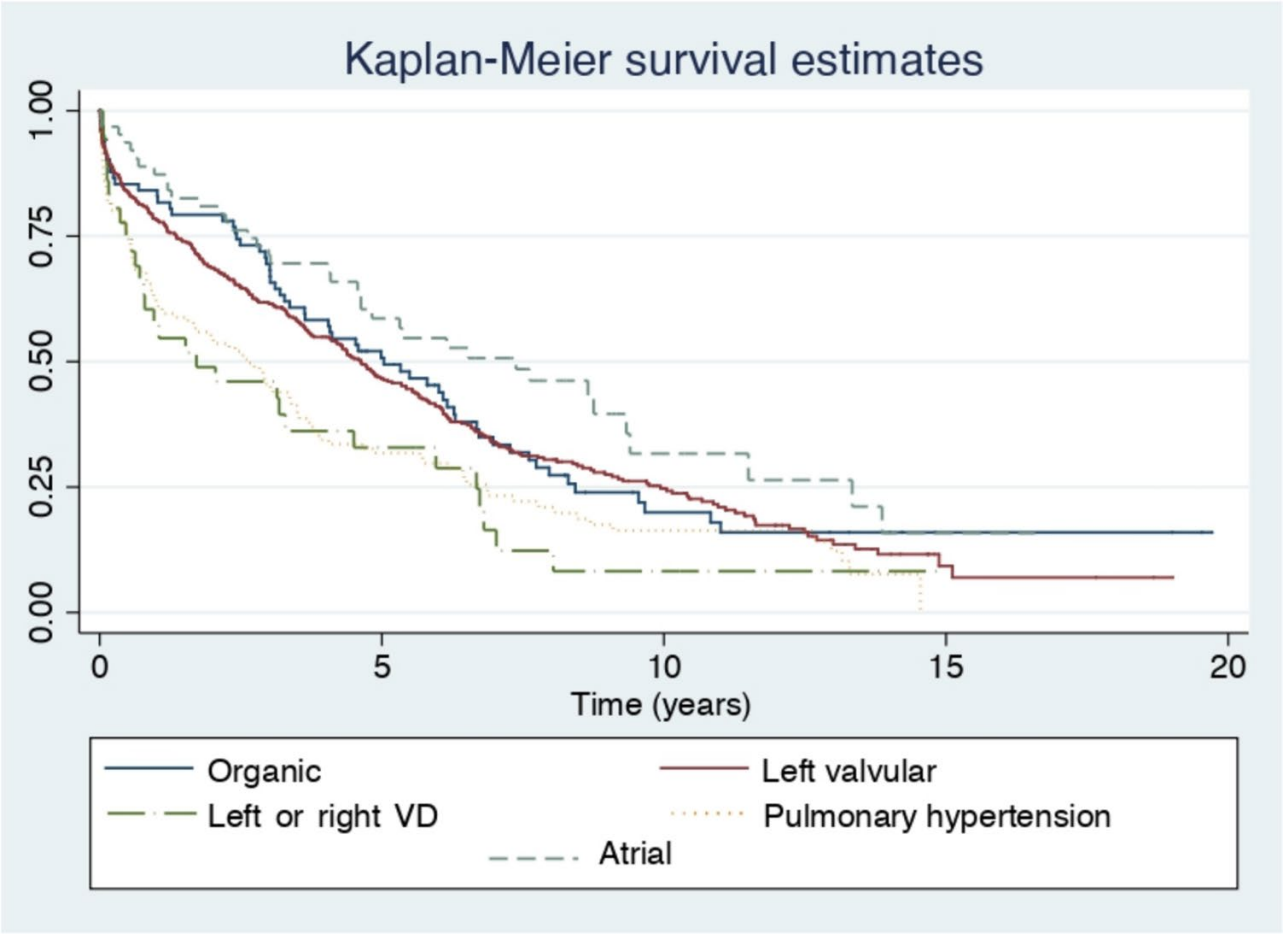
Kaplan–Meier survival curves for the five etiological groups

The overall incidence rate of mortality was 162.5 per 1000 patient-years (95% CI 148.7–176.2). When stratified by sex, the mortality rate was 242.5 per 1000 patient -years (95% CI 208.3–282.6) in men and 142.0 per 1000 patient-years (95% CI 128.3–157.1) in women. This sex difference in mortality was statistically significant, with an incidence rate ratio (IRR) between men and women of 1.7 (95% CI 1.4–2.1). Table 4 details incidence rates and rate ratios comparing each etiological group to the others combined. Across the entire sample, both TR associated with LV or RV dysfunction and TR associated with PH showed a higher risk of mortality events compared to the remaining etiological groups (IRR = 1, 95%CI: 1.1–2.5 and IRR = 1.5, 95% CI 1.2–1.9, respectively). Conversely, patients in the atrial TR group had a lower risk of events than those in the other groups (IRR = 0.6, 95% CI 0.4–0.9). This pattern was also observed when only analyzing women. However, in men, a higher event risk was noted only in the TR associated with PH group (IRR = 2.0, 95% CI 1.3–3.0). Table 5 breaks down the mortality data across the different etiological subgroups after the first year of follow-up. It can be observed that the differences between groups described for the entire follow-up period are already statistically significant after the first year. Figure 1 shows the Kaplan–Meier survival curves for the five etiological groups, with statistically significant differences observed between them (*p* < 0.001).

**Table 4:** Mortality incidence rate in each etiological group for the total sample and by sex

	Organic TR (*n* = 82)	Functional TR associated with left-sided valve disease (*n* = 432)	Functional TR associated with LV systolic dysfunction or primary RV disease (*n* = 39)	Functional TR associated with pulmonary hypertension (*n* = 139)	Atrial TR (*n* = 64)
*Total sample*					
Patient-years	446.5	1942.3	108.1	463.6	357.3
Events	62	302	29	108	38
Incidence rate^1^ (95% CI)	138.8 (108.3–178.1)	155.5 (138.9–174.0)	268.3 (186.5–386.1)	233.0 (192.9–281.3)	106.3 (77.4–146.1)
Incidence RR^2^ (95% CI)	0.8 (0.6–1.1)	0.9 (0.8–1.1)	**1.7 (1.1–2.5)**	**1.5 (1.2–1.9)**	**0.6 (0.4–0.9)**
*Male sex*					
Patient-years	97.6	379.6	60.5	66.0	72.6
Events	21	86	16	29	12
Incidence rate^1^ (95% CI)	215.1 (140.3–329.9)	226.6 (183.4–279.9)	264.7 (162.1–432.0)	439.4 (305.3–632.3)	165.3 (93,9–291.1)
Incidence RR^2^ (95% CI)	0.9 (0.5–1.4)	0.9 (0.6–1.2)	1.1 (0.6–1.8)	**2.0 (1.3–3.0)**	0.7 (0.3–1.2)
*Female sex*					
Patient-years	348.9	1562.7	47.6	397.6	284.8
Events	41	216	13	79	26
Incidence rate^1^ (95% CI)	117.5 (86.5–159.6)	138.2 (121.0–157.9)	272.9 (158.5–470.0)	198.7 (159.4–247.7)	91.3 (62.2–134.1)
Incidence RR^2^ (95% CI)	0.8 (0.6–1.1)	0.9 (0.8–1.2)	**2.0 (1.0–3.4)**	**1.5 (1.2–1.9)**	**0.6 (0.4–0.9)**

Statistically significant results are shown in bold (*p* < 0.05)

*CI* confidence interval, *LV* left ventricular, *RR* rate ratio, *RV* right ventricular, *TR* tricuspid regurgitation

^1^Per 1000 patient-years

^2^Comparison of each etiological group with the other groups combined

**Table 5:** Mortality incidence rate in each etiological group for the total sample after 1 year of follow-up

	Organic TR (*n* = 82)	Functional TR associated with left-sided valve disease (*n* = 432)	Functional TR associated with LV systolic dysfunction or primary RV disease (*n* = 39)	Functional TR associated with pulmonary hypertension (*n* = 139)	Atrial TR (*n* = 64)
*Total sample*					
Patient-months	825.5	4295.5	317.2	1223.0	703.7
Events	13	92	15	52	8
Incidence rate^1^ (95% CI)	15.2 (8.9–26.3)	21.4 (17.5–26.3)	47.3 (28.5–78.4)	42.5 (32.4–55.8)	11.4 (5.7–22.7)
Incidence RR^2^ (95% CI)	0.6 (0.3–1.0)	0.8 (0.6–1.0)	**2.0 (1.1–3.4)**	**2.0 (1.5–2.8)**	**0.4 (0.2–0.9)**

Statistically significant results are shown in bold (*p* < 0.05)

*CI* confidence interval, *LV* left ventricular, *RR* rate ratio, *RV* right ventricular, *TR* tricuspid regurgitation

^1^Per 1000 patient-months

^2^Comparison of each etiological group with the other groups combined

**Fig. 2:**
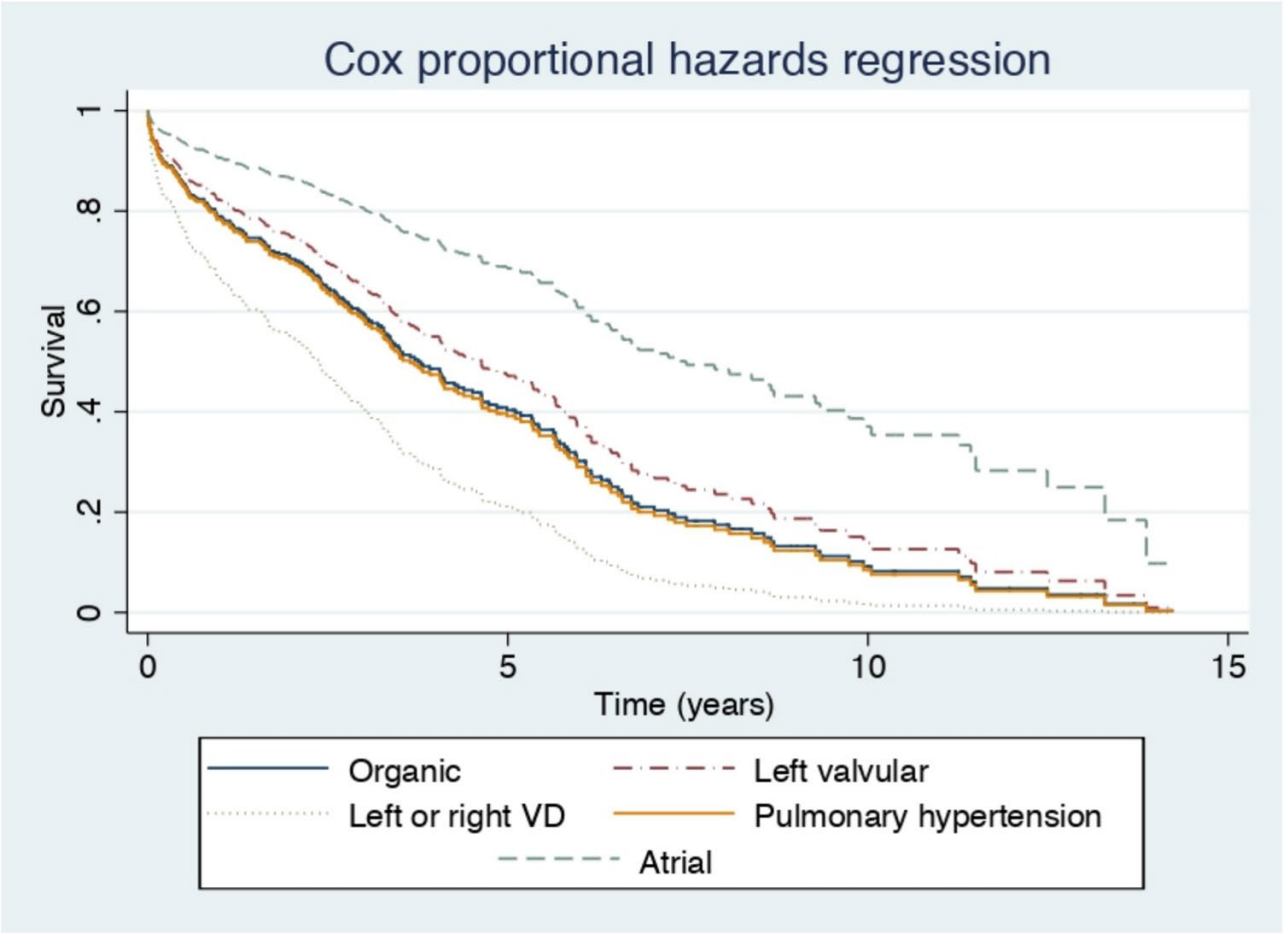
Adjusted survival curves according to this Cox Regression model

In the multivariable Cox regression model (Table 6), all etiological groups showed a higher mortality risk when compared to the atrial TR group: organic TR (aHR = 2.344, 95% CI 1.138–4.829), TR associated with left-sided valvular disease (aHR = 1.901, 95% CI 1.011–3.574), TR associated with LV systolic dysfunction or primary RV disease (aHR = 3.683, 95% CI 1.627–8.338), and TR associated with PH (aHR = 2.446, 95% CI 1.215–4.927). Among the covariates included in the final multivariable model, the following were independently associated with higher mortality: age (aHR = 1.037, 95% CI 1.020–1.055), male sex (aHR = 1.608, 95% CI 1.175–2.201), severe TR (aHR = 1.651, 95% CI 1.228–2.219), SPAP (aHR = 1.014, 95% CI 1.003–1.025), moderate LV hypertrophy (aHR = 2.518, 95% CI 1.464–4.331), chronic kidney disease (aHR = 1.578, 95% CI 1.166–2.135), and history of right HF (aHR = 1.580, 95% CI 1.061–2.353). Use of beta-blockers was inversely associated with mortality (aHR = 0.674, 95% CI 0.502–0.904). Figure 2 presents the adjusted survival curves according to this Cox regression model.

**Table 6:** Multivariable analysis of mortality using Cox regression

	Adjusted hazard ratio (95% CI)	*p*
*Etiology*		
Organic TR	2.344 (1.138–4.829)	0.021
Functional TR associated with left-sided valve disease	1.901 (1.011–3.574)	0.046
Functional TR associated with LV systolic dysfunction or primary RV disease	3.683 (1.627–8.338)	0.002
Functional TR associated with pulmonary hypertension	2.446 (1.215–4.927)	0.012
Atrial TR	1 (Reference)	
Age (years)	1.037 (1.020–1.055)	< 0.001
Sex (male)	1.608 (1.175–2.201)	0.003
*TR grade*		
Severe	1.651 (1.228–2.219)	0.001
More than severe	1.466 (0.641–3.353)	0.365
Moderate-severe	1 (Reference)	
SPAP (mmHg)	1.014 (1.003–1.025)	0.014
Previous cardiac surgery or valve intervention	1.268 (0.838–1.919)	0.262
*LV hypertrophy*		
Absent	1 (Reference)	
Mild	1.155 (0.839–1.589)	0.377
Moderate	2.518 (1.464–4.331)	0.001
Severe	0.773 (0.098–6.076)	0.870
RV dilation	1.335 (0.942–1.891)	0.104
RV dysfunction	0.980 (0.703–1.367)	0.905
Right atrial dilation	1.071 (0.707–1.622)	0.748
Diabetes	1.249 (0.930–1.679)	0.140
Chronic kidney disease	1.578 (1.166–2.135)	0.003
Liver disease	0.950 (0.584–1.546)	0.837
Lung disease	0.934 (0.646–1.530)	0.716
Ischemic heart disease	1.134 (0.755–1.659)	0.517
Previous hospitalization for heart failure	1.250 (0.886–1.763)	0.204
Hemoglobin < 11 g/dL	1.201 (0.887–1.627)	0.236
*NYHA functional class*		
I	1 (Reference)	
II	0.954 (0.482–1.885)	0.892
III	1.253 (0.619–2.536)	0.531
IV	1.565 (0.697–3.512)	0.278
Right heart failure	1.580 (1.061–2.353)	0.024
Diuretics	0.698 (0.472–1.032)	0.071
Beta-blockers	0.674 (0.502–0.904)	0.008

*CI* confidence interval, *LV* left ventricular, *NYHA* New York Heart Association, *RV* right ventricular, *SPAP* systolic pulmonary artery pressure, *TR* tricuspid regurgitation

## Discussion

In this study, we analyzed a multicenter series of patients with moderate-to-severe or greater TR, classified into five etiological groups. The results allowed us to describe the clinical characteristics and differences among these five TR groups. Furthermore, we compared the mortality across these groups, establishing that, when using the atrial TR group as a reference, the other etiologies presented a higher risk of mortality, regardless of the patients’ clinical or echocardiographic characteristics. Finally, we identified other mortality-related factors in these patients, some of which have been poorly described, such as beta-blocker therapy, or have shown controversy, such as male sex.

### Clinical characteristics of the five TR groups

In our sample, the most frequent category was functional TR associated with left-sided valvulopathy, showing a distribution pattern similar to that reported in other large series from various regions [1]. Female sex was the most frequent in the overall sample (71.2%) and in each TR category, except for the group with TR associated with left or right ventricular dysfunction (43.6% female). Many previous studies have described a predominance of women in large series of patients with significant TR of various etiologies [11–16]. Interestingly, a specific male predominance in TR associated with left ventricular dysfunction has been previously described [1, 4].

Regarding age, significant differences were also found among the TR categories in our sample, with the youngest group being those with TR associated with LV systolic dysfunction or primary RV disease, and the oldest group being those with atrial TR. We observed statistically significant differences in RV echocardiographic parameters, such as dilation and systolic dysfunction, with atrial TR being the least affected group, consistent with previous studies [1, 3, 17].

We also found statistically significant differences in comorbidities (NYHA classification, ischemic heart disease, and hospitalization for HF) and treatment (beta-blockers and aldosterone receptor antagonists) across the different TR etiological groups. Previous studies have suggested that patients with atrial TR present a less advanced functional class (III or IV) compared to the other etiological groups [18] or specifically to those with ventricular functional TR [3].

### Mortality comparison among the five groups

Our Cox regression model provided insights into the specific contribution of each TR group in predicting mortality, independent of the prognostic variables included in the adjustment. Using the atrial group as the reference, the aHR showed significant differences, with progressively higher risks for the following groups: (1) left-sided valvulopathy, (2) organic TR and TR secondary to PH (both groups with similar risks), and (3) LV systolic dysfunction or primary RV disease.

Identifying TR groups with prognosis specifically associated with their etiology is of great clinical importance as it may support the indication for early or late surgical or interventional treatment. Conversely, our study adds new findings to the limited previous evidence regarding the prognostic value specifically associated with each TR category. Galloo et al. found that in a multivariable mortality analysis, the predictors of death were TR secondary to left-sided heart disease, TR secondary to PH, and TR secondary to RV dysfunction, using atrial TR as the reference category [19]. We highlight three additional studies which have compared overall mortality across different TR categories using raw data but without adjusting for other predictors. Santoro et al. found the following increasing order of all-cause mortality: atrial TR < primary TR < TR secondary to left-sided valvulopathy < TR associated with pacemaker leads < TR secondary to LV systolic or diastolic dysfunction < TR secondary to precapillary PH [18]. Dietz et al. used primary TR as the reference and found a significant increase in risk for TR secondary to LV or secondary to left-sided valvulopathy [20]. Finally, Topilsky et al. ordered the groups in increasing mortality as follows: congenital TR < isolated or atrial TR < organic TR < TR secondary to PH < TR secondary to LV systolic dysfunction < TR secondary to left-sided valvulopathy [1]. However, our findings and those from the studies mentioned contrast with Rao et al., who analyzed a series of 2379 adults with newly diagnosed severe TR, comparing mortality (and other events) across three TR groups: primary TR, secondary TR, and TR associated with pacemaker leads. After adjusting for numerous clinical variables, they found no significant differences in mortality among the TR groups [21]. One of the main characteristics of Rao’s study is the grouping used for comparison. It is possible that a more detailed categorization, as performed in other studies, discriminates mortality better than a broader classification into three groups, where the secondary TR group includes a wide range of categories. Our findings on atrial TR mortality are also consistent with several previous studies that have shown a better prognosis for patients with atrial TR compared to those with ventricular TR [3, 17, 22].

### Other predictors of mortality during follow-up

Our study identified several variables associated with long-term mortality, many of which have been extensively reported in the literature: age [22, 23], the severity of TR [2, 13, 18], SPAP [12, 24], chronic kidney disease [22, 23], and right-sided HF [25]. A novel finding in our study is the protective effect of beta-blocker treatment. To our knowledge, no prior studies have specifically focused on assessing the effect of these drugs in patients with significant TR, limiting the current understanding of their role. Hochstadt et al. identified beta-blocker therapy as a protective factor for one-year mortality in patients with significant tricuspid regurgitation [26]. Conversely, other studies have failed to establish an association between beta-blocker use and mortality [27, 28]. The hypothesis we propose to explain the potential protective effect of beta-blockers is that their negative chronotropic activity could be beneficial. Several studies have demonstrated increased mortality in patients with severe TR and elevated resting heart rates above 80 bpm [29] or 90 bpm [30]. We believe that future studies should evaluate the prognostic value of beta-blockers, as well as heart rate and sinus rhythm control, specifically within different pathophysiological groups of TR to precisely determine the potential benefits of these therapeutic strategies.

Another significant finding from our study was the identification of male sex as a risk factor for mortality in these patients. The prognostic value of sex in severe TR remains controversial. Dietz et al. conducted a study specifically designed to assess differences in clinical characteristics and mortality between men and women with significant TR. In their cohort of 1569 patients, after matching both sexes using a propensity score, no significant differences in long-term survival were found [20]. Saed et al. evaluated 209 patients with significant TR to assess sex-related differences in RV hemodynamics and mortality, finding no significant differences in mortality either [31]. Several large series have assessed the prognostic role of sex in patients with TR of various etiologies, although this was not their primary focus. In most cases, sex was not identified as an independent predictor of mortality [23, 26, 32]. However, in some series, male sex was associated with an increased risk of adverse outcomes [19, 33]. A 2023 meta-analysis that included 12 studies with a total of 22,574 TR patients found a slightly higher long-term mortality risk for men compared to women (Risk Ratio = 1.16; 95% CI 1.06–1.26; *p* = 0.0007; *I*^2^ = 0%) [34]. Our study, in addition to identifying male sex as a predictor of mortality, reports mortality incidence rates for each sex across different etiological groups of TR, with seemingly disparate results (Table 4). The relatively small sample size in these groups prevents us from assessing whether sex was independently associated with mortality in any of these specific groups. We suggest that future studies explore the prognostic role of sex within each etiological group of TR, as the results may vary depending on the type of TR.

### Limitations

This study is based on a retrospective series involving three centers with different working protocols and data collected since 2002. Therefore, in addition to the inherent limitations of retrospective series, we must acknowledge that certain variables currently recognized as having prognostic value in significant TR could not be collected, as they were not recorded in many cases. The inclusion period varied significantly between centers, depending on the availability of an electronic database for patient identification. Nonetheless, including patients from centers of different levels (tertiary and secondary) enhances the external validity of our findings. The extensive inclusion period also led us to employ qualitative and semi-quantitative assessments for the degree of TR, which are recognized as less precise than quantitative assessments. For the same reason, a quantitative evaluation of RV size and function could not be performed, as done in other studies [24] which may have resulted in the assessed variables for the RV not being significantly associated with mortality. The classification of TR severity in our historical cohort could not be aligned with the currently accepted categories (severe, massive, and torrential TR). Various etiological classifications for TR have been proposed in the literature, and we based our classification on that described by Topilsky et al. [1]. This classification also has limitations, particularly in cases with multiple mechanisms responsible for TR. Finally, the analysis of the independent prognostic value of TR and the isolated prognostic value of each etiological group of TR in comparison with other clinical predictor variables could not be performed due to the absence of a control group without severe TR.

## Conclusions

This study analyzed the mortality of a multicenter series of patients with at least moderate to severe TR. Five etiological groups of TR were established, and after using atrial TR as a reference, the other four groups exhibited increased mortality after adjusting for multiple variables, with the group of TR secondary to LV dysfunction or primary RV disease showing the highest mortality. Various risk factors, such as male sex, and protective factors, such as beta-blocker treatment, were identified in relation to mortality. These findings suggest that analyzing etiology may be useful both in the clinical setting—improving risk estimation for patients with significant TR—and in research—facilitating the acquisition of solid evidence regarding the prognostic improvement associated with therapeutic tools used in TR management.
